# Genome sequence and overview of *Oligoflexus tunisiensis* Shr3^T^ in the eighth class *Oligoflexia* of the phylum *Proteobacteria*

**DOI:** 10.1186/s40793-016-0210-6

**Published:** 2016-12-13

**Authors:** Ryosuke Nakai, Takatomo Fujisawa, Yasukazu Nakamura, Tomoya Baba, Miyuki Nishijima, Fatma Karray, Sami Sayadi, Hiroko Isoda, Takeshi Naganuma, Hironori Niki

**Affiliations:** 1 Genetic Strains Research Center, National Institute of Genetics, 1111 Yata, Mishima, 411-8540 Japan; 2 Center for Information Biology, National Institute of Genetics, 1111 Yata, Mishima, 411-8540 Japan; 3 Department of Genetics, The Graduate University for Advanced Studies (SOKENDAI), 1111 Yata, Mishima, 411-8540 Japan; 4 Technical Department, TechnoSuruga Laboratory Co., Ltd., 330 Nagasaki, Shimizu-ku, Shizuoka, 424-0065 Japan; 5 Centre of Biotechnology of Sfax, University of Sfax, Route Sidi Mansour, km 6, BP 1177, 3018 Sfax, Tunisia; 6 Alliance for Research on North Africa (ARENA), University of Tsukuba, 1-1-1 Tennoudai, Tsukuba, 305-8572 Japan; 7 Graduate School of Biosphere Science, Hiroshima University, 1-4-4 Kagamiyama, Higashi-hiroshima, 739-8528 Japan

**Keywords:** *Oligoflexia*, *Proteobacteria*, RND-type efflux pump, Denitrification, Nitrous oxide (N_2_O)

## Abstract

*Oligoflexus tunisiensis* Shr3^T^ is the first strain described in the newest (eighth) class *Oligoflexia* of the phylum *Proteobacteria*. This strain was isolated from the 0.2-μm filtrate of a suspension of sand gravels collected in the Sahara Desert in the Republic of Tunisia. The genome of *O. tunisiensis* Shr3^T^ is 7,569,109 bp long and consists of one scaffold with a 54.3% G + C content. A total of 6,463 genes were predicted, comprising 6,406 protein-coding and 57 RNA genes. Genome sequence analysis suggested that strain Shr3^T^ had multiple terminal oxidases for aerobic respiration and various transporters, including the resistance-nodulation-cell division-type efflux pumps. Additionally, gene sequences related to the incomplete denitrification pathway lacking the final step to reduce nitrous oxide (N_2_O) to nitrogen gas (N_2_) were found in the *O. tunisiensis* Shr3^T^ genome. The results presented herein provide insight into the metabolic versatility and N_2_O-producing activity of *Oligoflexus* species.

## Introductions

The phylum *Proteobacteria* traditionally comprises five classes of *Alphaproteobacteria*, *Betaproteobacteria*, *Gammaproteobacteria*, *Deltaproteobacteria* and *Epsilonproteobacteria* [1, 2], with two additional classes ‘*Zetaproteobacteria*’ and *Acidithiobacillia* proposed by Emerson et al. [3] and Williams and Kelly [4], respectively. *Proteobacteria* hosts the greatest number of isolates and sequenced genomes among the prokaryotic phyla [5] and contains members exhibiting extremely diversified metabolisms relevant to global carbon, nitrogen, and sulfur cycles [2]. This phylum recently gained the eighth (or seventh if yet-to-be-validated ‘*Zetaproteobacteria*’ is excluded) class *Oligoflexia* with the cultured species *Oligoflexus tunisiensis* type strain Shr3^T^ [6]. The class *Oligoflexia* includes environmentally-derived 16S rRNA gene sequences, otherwise known as environmental clones or phylotypes, recovered from a variety of habitats including soils, the Taklamakan Desert, glacial ice, lake water, seawater, human skin, and the guts of earthworms [6]. In contrast to their wide distribution, *Oligoflexia*-affiliated clones have rarely been found in clone libraries [7]; accordingly, it has been suggested that the *Oligoflexia* members show a small population size, belonging to the so-called rare biosphere [8].

At the time of writing, *O. tunisiensis* Shr3^T^ was the only cultured species within the class *Oligoflexia*. Physiological and biochemical features of strain Shr3^T^ could not be fully characterized because of restrictive culture conditions owing to the slow-growing nature of this strain [6]. The phenotypic information is essential for understanding its ecological role and biotechnological potentials. Here, we compensated for the limited knowledge regarding *Oligoflexia* members by conducting genomic analysis of strain Shr3^T^.

## Organism information

### Classification and features

During a study of ultramicro-sized bacteria that could pass through 0.2-μm pore-size filters, which are generally used for sterile filtration to remove microorganisms, we isolated the bacterium designated isolate Shr3 [9]. The isolation source of this bacterium was a 0.2-μm filtrate of the suspension of sand gravels collected in December 2008 in Matmata (33° 31’ N 9° 57’ E) on the eastern margin of the Sahara Desert in the Republic of Tunisia. Isolate Shr3 was thereafter described as the type strain of *Oligoflexus tunisiensis*, the first cultured representative of the novel class *Oligoflexia* [6].

**Fig. 1:**
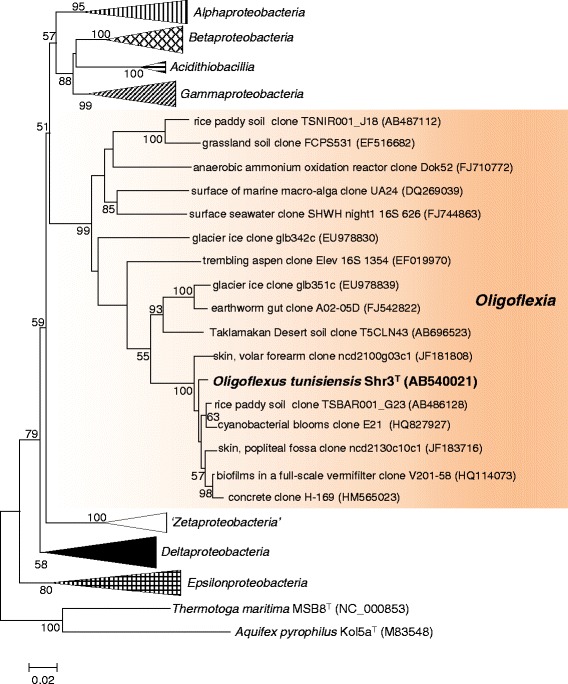
Phylogenetic relationships between *O. tunisiensis* Shr3^T^ and related environmental clones in the phylum *Proteobacteria* based on 16S rRNA gene sequences. At the time of writing, strain Shr3^T^ was the only cultured species within the class *Oligoflexia*. The tree, generated with MEGA 6.0 [34] using the neighbor-joining method [35], is based on a comparison of approximately 1130 nucleotides. Bootstrap values >50%, expressed as percentages of 1000 replicates, are shown above and below branches. Bar: 0.02 substitutions per nucleotide position

Figure 1 shows the phylogenetic position of *O. tunisiensis* and related environmental clones in a 16S rRNA-based evolutionary tree. The sequence of the three 16S rRNA gene copies in the genome was 100% identical to the previously published 16S rRNA gene sequence (DDBJ/EMBL/GenBank accession no. AB540021 [6]). The database search showed that seven environmental clones had a >97% high similarity with the *O. tunisiensis* 16S rRNA gene sequence [7]. The seven clones were from rice paddy soil, cyanobacterial blooms in a hypereutrophic lake, a microalgal photobioreactor, a bio-filter, and human skin [7]. Strain Shr3^T^ has been deposited in the Japan Collection of Microorganisms and the National Collection of Industrial, food and Marine Bacteria under accession numbers JCM 16864^T^ and NCIMB 14846^T^, respectively. The general features of strain Shr3^T^ are reported in Table 1.

**Table 1:** Classification and general features of *Oligoflexus tunisiensis* type strain Shr3^T^ according to MIGS standards [30]

MIGS ID	Property	Term	Evidence code^a^
	Classification	Domain *Bacteria*	TAS [31]
		Phylum *Proteobacteria*	TAS [32]
		Class *Oligoflexia*	TAS [6]
		Order *Oligoflexales*	TAS [6]
		Family *Oligoflexaceae*	TAS [6]
		Genus *Oligoflexus*	TAS [6]
		Species *Oligoflexus tunisiensis*	TAS [6]
		Type strain: Shr3^T^	TAS [6]
	Gram stain	negative	TAS [6]
	Cell shape	filamentous-shaped	TAS [6, 7]
	Motility	non-motile	TAS [6]
	Sporulation	none	TAS [6]
	Temperature range	20–37 °C	TAS [6]
	Optimum temperature	25–30 °C	TAS [6]
	pH range; Optimum	7.0–9.5; 7.0–8.0	TAS [6]
	Carbon source	heterotrophic	TAS [6]
MIGS-6	Habitat	desert	TAS [6]
MIGS-6.3	Salinity	0–0.5% (w/v) NaCl	TAS [6]
MIGS-22	Oxygen requirement	aerobic	TAS [6]
MIGS-15	Biotic relationship	free-living	TAS [6]
MIGS-14	Pathogenicity	not reported	
MIGS-4	Geographic location	Matmata, Republic of Tunisia	TAS [6]
MIGS-5	Sample collection	December 2008	TAS [6]
MIGS-4.1	Latitude	33.53	TAS [6]
MIGS-4.2	Longitude	9.96	TAS [6]
MIGS-4.4	Altitude	not determined	

^a^Evidence codes – IDA: Inferred from Direct Assay; TAS: Traceable Author Statement (i.e., a direct report exists in the literature); NAS: Non-traceable Author Statement (i.e., not directly observed for the living, isolated sample, but based on a generally accepted property for the species, or anecdotal evidence). These evidence codes are from the Gene Ontology project [33]

**Fig. 2:**
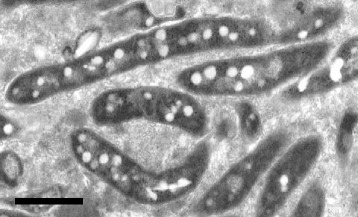
Transmission electron micrograph of *O. tunisiensis* Shr3^T^. Many low electron-density particles were observed. Cells were grown on R2A medium for 7 days at 25 °C. Scale: 1 μm

*O. tunisiensis* Shr3^T^ is a Gram-negative, aerobic, non-motile, filamentous bacterium of 0.4–0.8 μm in width when cultivated under the experimental culture conditions [6]. Some cells exhibited a spiral, spherical (or curled), or curved rod morphology [7]. Although the factors controlling the cell shapes are still unclear, the morphological flexibility is likely associated with their ability to pass through 0.2-μm filters. Strain Shr3^T^ grows in the R2A medium [6]. The cells showed slow growth, with 3–5 days required before colonies could be seen by the naked eye [6]. The growth occurs at NaCl concentrations <1.0% (w/v), 20–37 °C (optimum 25–30 °C), and pH 7.0–9.5 (optimum pH 7.0–8.0) [6]. Enzyme activities of esterase lipase, leucine arylamidase, trypsin, naphthol-AS-BI-phosphohydrolase and α-mannosidase are positive [6]. Transmission electron microscopy revealed that cells contained many low electron-dense particles (Fig. 2). Some, but not all, particles were stained by Sudan black B upon staining PHB or lipophilic particles. Because cells swollen by accumulated PHB were not observed when grown on PHB-containing medium [6], the particles stained with Sudan black B are likely lipophilic granules.

#### Chemotaxonomy

The major respiratory quinone was menaquinone-7 (MK-7) [6]. The dominant cellular fatty acids were C_16 : 1_ *ω*5c (65.7%) and C_16 : 0_ (27.5%), the major hydroxy fatty acid was C_12 : 0_ 3-OH (1.3%), and the minor fatty acids included C_10:0_, C_12:0_, C_15:0_, C_17:0_, C_18:0_ and C_18:1_ *ω*5c [6]. The fatty acid, C_16 : 1_ *ω*5c, was also detected in myxobacteria of *Cystobacterineae* in the class *Deltaproteobacteria*, but at only 15–39% [10].

## Genome sequencing information

### Genome project history

Phenotypic features of strain Shr3^T^ are described above, but could not be fully tested because of restrictive culture conditions [6]. Therefore, this organism was selected for genome sequencing to investigate the basis of its ecological role and biotechnological potentials. The genome project is deposited in the Genomes OnLine Database [11] under the accession number Gp0139475. The information genome sequence is available from the DDBJ/EMBL/GenBank database. A summary of this genome project is shown in Table 2.

**Table 2:** Project information

MIGS ID	Property	Term
MIGS 31	Finishing quality	High-quality draft
MIGS-28	Libraries used	Pair-end library and mate-pair library
MIGS 29	Sequencing platforms	Illumina HiSeq 2000
MIGS 31.2	Fold coverage	149 ×
MIGS 30	Assemblers	SOAPdenovo version 2.04
MIGS 32	Gene calling method	Prodigal
	Locus Tag	Ga0118670 (IMG-ER)
	GenBank ID	BDFO01000001
	GenBank Date of Release	30 June 2016
	GOLD ID	Gp0139475
	BIOPROJECT	PRJDB4872
MIGS 13	Source Material Identifier	JCM 16864, NCIMB 14846
	Project relevance	ecology, biotechnology

### Growth conditions and genomic DNA preparation

A culture of *O. tunisiensis* Shr3^T^ grown aerobically in R2A broth (DAIGO; Nihon Pharmaceutical Co., Ltd., Tokyo, Japan) at 30 °C was used to prepare genomic DNA. The genomic DNA was extracted using Qiagen Genomic-Tip 500/G columns according to the manufacturer’s instructions. The quantity and purity of the extracted DNA was checked by spectrophotometric measurement at 260 nm and agarose gel electrophoresis.

### Genome sequencing and assembly

The genome sequence was generated using paired-end sequencing (2 × 90 bp) on an Illumina HiSeq 2000 platform at the BGI with the pair-end library and mate-pair library of two different insert sizes, 456 to 496 bp and 6310 to 6350 bp. After trimming of low quality reads, 1130 Mb was obtained and assembled into 19 contigs in one scaffold using SOAPdenovo version 2.04 [12]. The assembly result was locally optimized according to the paired-end and overlap relationship via mapping reads to obtained contigs. A summary of this genome sequence is shown in Table 3.

**Table 3:** Genome statistics

Attribute	Value	% of Total
Genome size (bp)	7,569,109	100.00
DNA coding (bp)	6,849,121	90.49
DNA G + C (bp)	4,113,347	54.34
DNA scaffolds	1	100.00
Total genes	6,463	100.00
Protein coding genes	6,406	99.12
RNA genes	57	0.88
Pseudogenes	not determined	not determined
Genes in internal clusters	1,494	23.12
Genes with function prediction	4,051	62.68
Genes assigned to COGs	2,938	45.46
Genes with Pfam domains	4,268	66.04
Genes with signal peptides	1,084	16.77
Genes with transmembrane helices	1,393	21.55
CRISPR repeats	8	

### Genome annotation

Gene sequences were identified via the Prodigal V2.6.3 [13] as part of the DOE-JGI genome annotation pipeline in the Integrated Microbial Genomes–Expert Review (IMG-ER) system [14]. Gene functional annotation as well as data visualization was conducted within the IMG-ER [15]. The predicted coding sequences were translated and used to search the National Center for Biotechnology Information non-redundant, UniProt, TIGR-Fam, Pfam, KEGG, COG, and InterPro databases. Identification of RNA gene sequences and miscellaneous features were carried out using HMMER 3.1b2 [16] and INFERNAL 1.0.2 and 1.1.1 [17]. Additional functional prediction was performed with the RAST server [18] under accession number 708132.3. Candidate CRISPR regions were detected using the CRISPRFinder program [19].

### Genome Properties

**Fig. 3:**
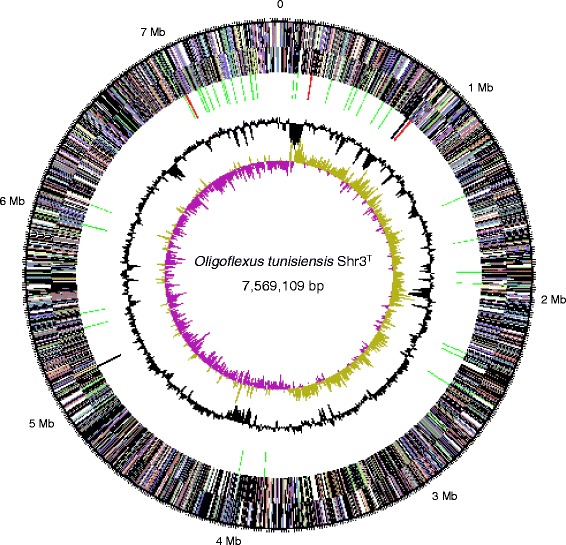
Graphical circular map of the chromosome of *O. tunisiensis* Shr3^T^. From outside to the center: genes on forward strand (color by COG categories), genes on reverse strand (color by COG categories), RNA genes (tRNAs green, rRNAs red, other RNAs black), GC content, GC skew

The genome of *O. tunisiensis* Shr3^T^ consists of a 7,569,109 bp long chromosome with a 54.3% G + C content (Table 3). Of the 6463 predicted genes, 6406 were protein-coding genes and 57 were RNA genes (three rRNA operons, 46 tRNAs, and two miscRNAs). The majority of the protein-coding genes (62.7%) were assigned to a putative function. The remaining ones were annotated as hypothetical proteins. The distribution of genes classified into COGs functional categories is shown in Table 4 and Fig. 3.

**Table 4:** Number of genes associated with general COG functional categories

Code	Value	%age	Description
J	228	6.91	Translation, ribosomal structure and biogenesis
A	1	0.03	RNA processing and modification
K	154	4.67	Transcription
L	103	3.12	Replication, recombination and repair
B	1	0.03	Chromatin structure and dynamics
D	31	0.94	Cell cycle control, Cell division, chromosome partitioning
V	87	2.64	Defense mechanisms
T	314	9.52	Signal transduction mechanisms
M	229	6.94	Cell wall/membrane biogenesis
N	125	3.79	Cell motility
U	44	1.33	Intracellular trafficking and secretion
O	159	4.82	Posttranslational modification, protein turnover, chaperones
C	172	5.21	Energy production and conversion
G	142	4.30	Carbohydrate transport and metabolism
E	264	8.00	Amino acid transport and metabolism
F	73	2.21	Nucleotide transport and metabolism
H	170	5.15	Coenzyme transport and metabolism
I	193	5.85	Lipid transport and metabolism
P	156	4.73	Inorganic ion transport and metabolism
Q	100	3.03	Secondary metabolites biosynthesis, transport and catabolism
R	337	10.22	General function prediction only
S	153	4.64	Function unknown
-	3,525	54.54	Not in COGs

The total is based on the total number of protein coding genes in the genome

## Insights from the genome sequence

The genome of *O. tunisiensis* Shr3^T^ encoded genes for ABC transporters of amino acid, oligopeptide/dipeptide, and phosphonate, ammonium and nitrate/nitrite transporters, as well as RND-type efflux pumps. One of the amino acid sequences (Ga0118670_114686) classified as an RND pump showed a high similarity (67% identity and 99% coverage) to sequences of the pathogenic bacteria *Achromobacter xylosoxidans* and *Pseudomonas aeruginosa*. The RND-type efflux system is widely distributed in Gram-negative bacteria and known to promote resistance to various kinds of antimicrobial substances, termed as multidrug resistance [20].

In support of its aerobic growth, gene sequences assigned to different terminal oxidases including *aa*_3_- and *cbb*_3_-type cytochrome *c* oxidases (COG0843 and COG3278) and cytochrome *bd*-type quinol oxidase (COG1271 and COG1294) were found in the Shr3^T^ genome.

The Shr3^T^ genome contained a *nirK* gene coding for a copper-dependent nitrite reductase (Nir) (Ga0118670_114712) involved in denitrification, a major component of the nitrogen cycle [21]. Denitrification is the dissimilatory reduction of nitrate or nitrite to nitrogen gas (NO_3_^−^ → NO_2_^−^ → NO → N_2_O → N_2_) [22] that usually occurs under oxygen-limiting conditions [21]. The key steps releasing gaseous products NO, N_2_O, and N_2_ are catalyzed by Nir, nitric-oxide reductase (Nor) and nitrous oxide reductase (Nos), respectively [23, 24]. There are two structurally different nitrite reductases among denitrifiers: a copper-containing type (Cu-Nir) encoded by the *nirK* gene and a cytochrome *cd*_1_-containg one (*cd*_1_-Nir) encoded by the *nirS* gene [24]. The *nirS* gene was absent from the *O. tunisiensis* Shr3^T^ genome.

The NirK deduced amino acid sequence of *O. tunisiensis* Shr3^T^ was most closely related to that of *Bdellovibrio bacteriovorus* of the class *Deltaproteobacteria*, with 70% identity and 96% coverage. *B. bacteriovorus* has an incomplete denitrifying pathway with a Cu-Nir, a cytochrome *c*–dependent Nor (cNor), and no Nos [25, 26]. *O. tunisiensis* Shr3^T^ also had a partial pathway containing the Cu-Nir described above, a quinol-dependent Nor (qNor), and no Nos inferred from the genome data. Strain Shr3^T^ has two copies of the gene encoding qNor (Ga0118670_112818 and Ga0118670_114769). NorR protein is known to regulate Nor expression in response to NO [27, 28]. The transcription regulator *norR* gene (Ga0118670_114771) was nearly adjacent to one of two copies of the qNor-encoding gene in the genome.

Our results suggest that the *Oligoflexus* species has the capability to produce N_2_O as a final product of the incomplete denitrification lacking the last step (reduction of N_2_O to N_2_). N_2_O is known as a strong greenhouse gas, as well as an ozone-depleting substance [29]. Accordingly, future studies should examine the N_2_O-producing phenotype of strain Shr3^T^.

## Conclusions

In this study, we characterized the genome of *O. tunisiensis* Shr3^T^, the first cultured representative of the novel proteobacterial class *Oligoflexia*. The genome sequence gives us insight into the metabolic versatility and incomplete denitrification pathway of *Oligoflexus* species. The genome information will facilitate future systematics and comparative genomics studies within the phylum *Proteobacteria*.

## Abbreviations

PHB: Polyhydroxybutyrate
RND: Resistance-nodulation-cell division
